# Artificial intelligence-based immunoprofiling serves as a potentially predictive biomarker of nivolumab treatment for advanced hepatocellular carcinoma

**DOI:** 10.3389/fmed.2022.1008855

**Published:** 2022-11-08

**Authors:** Jan-Mou Lee, Yi-Ping Hung, Kai-Yuan Chou, Cheng-Yun Lee, Shian-Ren Lin, Ya-Han Tsai, Wan-Yu Lai, Yu-Yun Shao, Chiun Hsu, Chih-Hung Hsu, Yee Chao

**Affiliations:** ^1^FullHope Biomedical Co., Ltd., New Taipei City, Taiwan; ^2^Department of Oncology, Taipei Veterans General Hospital, Taipei, Taiwan; ^3^School of Medicine, College of Medicine, National Yang Ming Chiao Tung University, Taipei, Taiwan; ^4^College of Medicine, Graduate Institute of Oncology, National Taiwan University, Taipei, Taiwan; ^5^Department of Medical Oncology, National Taiwan University Cancer Center, Taipei, Taiwan; ^6^Department of Oncology, National Taiwan University Hospital, Taipei, Taiwan

**Keywords:** hepatocellular carcinoma (HCC), immunoprofiling, predictive biomarker, nivolumab (PubChem SID: 178103907), immunotherapy, artificial intelligence

## Abstract

Immune checkpoint inhibitors (ICI) have been applied in treating advanced hepatocellular carcinoma (aHCC) patients, but few patients exhibit stable and lasting responses. Moreover, identifying aHCC patients suitable for ICI treatment is still challenged. This study aimed to evaluate whether dissecting peripheral immune cell subsets by Mann-Whitney U test and artificial intelligence (AI) algorithms could serve as predictive biomarkers of nivolumab treatment for aHCC. Disease control group carried significantly increased percentages of PD-L1^+^ monocytes, PD-L1^+^ CD8 T cells, PD-L1^+^ CD8 NKT cells, and decreased percentages of PD-L1^+^ CD8 NKT cells via Mann-Whitney U test. By recursive feature elimination method, five featured subsets (CD4 NKTreg, PD-1^+^ CD8 T cells, PD-1^+^ CD8 NKT cells, PD-L1^+^ CD8 T cells and PD-L1^+^ monocytes) were selected for AI training. The featured subsets were highly overlapping with ones identified via Mann-Whitney U test. Trained AI algorithms committed valuable AUC from 0.8417 to 0.875 to significantly separate disease control group from disease progression group, and SHAP value ranking also revealed PD-L1^+^ monocytes and PD-L1^+^ CD8 T cells exclusively and significantly contributed to this discrimination. In summary, the current study demonstrated that integrally analyzing immune cell profiling with AI algorithms could serve as predictive biomarkers of ICI treatment.

## Introduction

T cell checkpoint blockade immunotherapies targeting CTLA-4 and the axis of PD-1/PD-L1 by therapeutic antibodies have revolutionized cancer treatment following the clinical success achieved (1). PD-1 inhibitors, such as nivolumab and pembrolizumab, got accelerated approval from the Food and Drug Administration (FDA) of the United States in treating advanced hepatocellular carcinoma (aHCC) patients after sorafenib failure based on two phase-II global open-label clinical trials, Checkmate-040 and Keynote-224 (2, 3). Later, the FDA approved a combination of bevacizumab and atezolizumab (a PD-L1 inhibitor) as the first-line treatment for aHCC patients based on the phase-III randomized control trial, Imbrave-150 (4). The objective response rates in the three ICIs are 15% (nivolumab), 18% (pembrolizumab) and 27.3% (atezolizumab), respectively. The corresponding median progression-free survival of the three ICIs is 4.1 months (nivolumab), 7.0 months (pembrolizumab), and 6.8 months (atezolizumab).

Despite considerable advancements in the above-mentioned clinical studies, epidemiologic data and ongoing clinical trials suggest that most patients receiving immune checkpoint inhibitors (ICI) do not get clinical benefits from ICIs (5). Corresponding cellular and molecular mechanisms underlying the diversity of disease responses are multifactorial and still not completely understood (6). Therefore, exploring predictive biomarkers annealing with immune cell responses for ICIs treatment in aHCC emerged as a challenging unmet need.

Flow cytometry allows a detailed single-cell characterization of adaptive and innate immune landscapes, thereby providing a unique platform to discriminate immune cell subsets that can be exploited in an immunotherapeutic setting (7). Previously, we conducted an observational study to screen the immune cell subsets of aHCC patients and identified a significantly lower percentage of PD-1^+^ B cells in peripheral blood mononuclear cells (PBMCs) from aHCC patients with disease progression responses than those with disease control response after nivolumab treatment (8, 9). In a pilot study of metastatic melanoma, CD14^+^CD16^–^HLA-DR*^hi^* monocyte content strongly correlated with disease overall survival (OS) in ICI-treated patients (10). This study encouraged us to enroll more aHCC subjects and re-analyze immunoprofiling from aHCC patients with disease control and disease progression responses after nivolumab treatment. Furthermore, by automatically discovering integrated patterns from sophisticated biomedical data (11), machine learning (ML) has been applied in identifying biomarkers for predictive drug responses and disease diagnoses such as cancers (12, 13). Accordingly, the current study aimed to re-compare the immunoprofiling between aHCC patients with disease control and disease progression responses to determine the immune cell subsets regarding the efficacy of nivolumab. Additionally, we attempted to preliminarily investigate whether analyzing immunoprofiling of aHCC by ML can predict clinical responses before nivolumab treatments.

## Materials and methods

### Study design

This study was an extension of our previous one with two changes; additional seven patients were included, and the repertoire of analyzed immune cell subsets was modified from 55 to 50 (8). The trial protocol complied with the Declaration of Helsinki and was reviewed and approved by the Institutional Review Board of Taipei Veteran General Hospital (approval code 2018-08-017AC). We enrolled aHCC patients who were also subjects of the Checkmate-040 trial from National Taiwan University Hospital (Taipei, Taiwan) and Taipei Veteran General Hospital (Taipei, Taiwan). Eligible criteria included: (1) older than 20 years old, (2) histological confirmed aHCC based on the practical guideline, (3) Child-Pugh class A, (4) Eastern Cooperative Oncology Group (ECOG) performance status 0 or 1, (5) adequate organ function for Checkmate-040 trial, (6) plan to receive nivolumab treatment, (7) no therapeutic modalities within four weeks prior to initial dose of nivolumab, and (8) did not receive any immuno-modulating medications. Patients who did not sign the informed consent form were ineligible. After signing the informed consent form, we collected 20 mL of peripheral blood from the patients before 3 mg/kg of nivolumab treatment and recorded their clinical outcomes as treatment finished based on RECIST version 1.1.

### Reagents and antibodies

Ficoll-Paque™ medium (Ficoll, density 1.077 g/mL, Cytiva 17544202, Marlborough, MA, USA), bovine serum albumin (BSA, Sigma-Aldrich A7030, Merck KGaA, Darmstadt, Germany), and sodium azide (NaN_3_, Sigma-Aldrich S2002, Merck) were applied for peripheral blood mononuclear cells (PBMCs) isolation. FoxP3/Transcription factor staining buffer set (Thermo-Fisher 00-5523-00, Waltham, MA, USA) was used in immunostaining of intracellular markers.

A total of sixteen fluorescent-labeled antibodies were used to identify specific immune cell subsets. The antibodies enlisted in the following were obtained from Beckman-Coulter (Brea, CA, USA): Allophycocyanin/Alexa Fluor 700 (APC/AF700)-conjugated anti-CD56 (N901, B10822), APC/Alexa Fluor 750 (APC/AF750)-conjugated anti-CD14 (RMO52, A86052) and anti-CD19 (J3-119, A78838), Krome orange (KO)-conjugated anti-CD3 (UCHT1, B00068) and anti-CD8 (B9.11, B00067), and phycoerythrin/cyanine 5.5 (PE/Cy5.5)-conjugated anti-CD4 (SFCI12T4D11, 6607101). Following antibodies were obtained from Biolegend (San Diego, CA, USA): APC-conjugated anti-CD11c (3.9, 301614) and anti-TCRγ/δ (236A/E7, 331212), fluorescein isothiocyanate-conjugated anti-TCRα/β (L3D10, 306705), PB-conjugated anti-CD69 (FN50, 310919), PE-conjugated anti-CD25 (BC96, 302606), and anti-PD-L1 (B1, 329706), and peridinin-chlorophyll-protein/Cy5.5 (PerCP/Cy5.5)-conjugated anti-PD-1 (IP26, 329914). APC-conjugated anti-FoxP3 (29E.2A3, 17-4777-42) antibody was purchased from Thermo-Fisher. All antibodies were aliquoted as received and stored under recommended conditions until use.

### Isolation of peripheral blood mononuclear cells

Isolating protocol of PBMCs was identical to that in our previous study (8). Briefly, peripheral blood was mixed with an aliquot of phosphate buffer saline (PBS) and loaded into Ficoll-preloaded conical tubes. Then, tubes were centrifuged for 30 min with 400× *g* and without brake. PBMCs were collected from the buffy coat, washed twice with PBS, and suspended in staining buffer (0.5% BSA/0.02% NaN_3_/ PBS) for immunostaining.

### Immunostaining and fluorescent determination of peripheral blood mononuclear cells

Isolated PBMCs were incubated with staining buffer containing targeted antibodies for 30 min under 4*^o^*C and dark environment. Afterward, stained PBMCs were divided into two: one was determined fluorescence using a flow cytometer (Navios, Beckman-Coulter), and another was fixed and permeabilized by FoxP3/transcription factor staining buffer kit, stained with anti-FoxP3 antibody, and determined fluorescence by flow cytometer.

### Data acquisition and machine learning

Data collection from the flow cytometer and identification of immune cell subsets were performed by Kaluza analysis software V1.3 (Beckman-Coulter). A total of fifty immune cell subsets were identified based on their phenotypes described in Supplementary Table 1 through a sequentially gating process shown in Supplementary Figures 1–4. After gating, the abundance of immune cell subsets was recorded, used in ML, and tested the statistical difference between patients with disease control responses and those with disease progression responses.

The schematic illustration of the ML process was shown in Figure 2. Three ML algorithms applied in this study were random forest classifier (14), logistic regression (15), and support vector machines (16). In brief, we constructed a dataset that comprised the abundance of immune cell subsets from each subject. Then, RFECV was used to select featured subsets for separating aHCC patients with disease control responses from those with disease progression responses from the dataset according to the ranking of feature importance of the machine learning model (17). RFECV starts with full features, then recursively removes the weakest one until the model develops poor performance. We apply Random Forest (RF) algorithm to RFECV as its classifier that helps to add randomness to subset selections and to offer final predictions based on the majority voting. Due to the smaller size of the dataset, we use Leave-One-Out Cross-Validation (LOOCV) in RFECV to ensure that each specimen has an opportunity to represent the entire validation set, thus providing a more robust estimate of model performance. We also take the weighted average F1 score as the evaluation metric of RFECV, which is calculated by finding the mean of the F1-score through the support of each class. Subsequently, the featured subsets were applied for ML model training with the LOOCV in cross-validation and hyperparameters enlisted in Supplementary Table 2. After model training, the performance of ML models in distinguishing aHCC patients with disease control responses from those with disease progression responses was assessed by receiver operating characteristic (ROC) analysis. Additionally, the contribution of featured immune cell subsets in each ML model was weighed by SHapley Additive exPlanations (SHAP). All packages applied in ML were open-sourced and available on Github: RFECV, min-max scaling, random forest classifier, logistic regression, support vector machines, leave-one-out validator, and ROC were included in the scikit-learn kit (download link: https://github.com/scikit-learn/scikit-learn); Optuna^1^ and SHAP were independent packages.^2^

### Statistical analysis

The comparison of immunoprofiling between aHCC patients with disease control responses and those with disease progression responses was shown in mean ± standard deviation (SD) by Prism V9.0 (GraphPad Software, San Diego, CA, USA). Mann-Whitney U test was used to analyze the statistical significance between patients with disease control response and those with disease progression response which comparisons with statistical significance were labeled with * or ^**^ as *p* < 0.05 or 0.01.

## Results

### Baseline characteristic of the subjects

We grouped enrolled patients into disease control and disease progression groups based on their clinical response to nivolumab and described their demographics in Table 1. Briefly, 15 (15 male and 0 female) and 8 (6 male and 2 female) patients were grouped into disease control and disease progression groups whose age were comparable. All patients were diagnosed with Barcelona Clinic Liver Cancer stage C and Child-Pugh class A. Six patients in disease control group and three patients in disease progression group exhibited portal vein invasion. Twenty-two patients showed distal metastasis. Median contents of α-fetoprotein of disease control and disease progression were 2262 ng/mL and 124 ng/mL, respectively. Fourteen patients (61%) and four patients (17%) had positive results in hepatitis B viral antigen assay and anti-hepatitis C virus antibodies, respectively, which fit the high prevalence of hepatitis B and hepatitis C viral infections in Taiwan (18). After nivolumab treatment, five patients showed partial responses, ten with stable diseases, and eight with disease progression. Median progression-free survival of two groups were 4.0 months (disease control group) and 1.4 months (disease progression group), respectively. We merged the data of immunoprofiling from patients with partial responses and stable disease into the disease control group and applied them in the following analysis.

**TABLE 1:** Demography of the enrolled subjects.

	Disease control group	Disease progression group	*p*
Total	15	8	
Median age (range, in y)	63 (38–68)	62 (41–75)	0.2912
Gender (male/total, n)	15/15	6/8	
Child-Pugh class (A/total, n)	15/15	8/8	
BCLC (B/C, n)	0/15	0/8	
Portal vein thrombosis (positive/total, n)	6/15	3/8	
Distal metastasis (positive/total, n)	14/15	8/8	
Median AFP (range, in ng/mL)	2262 (14 – 69195)	124 (2 – 17866)	0.0695
HBsAg (positive/total, n)	8/15	6/8	
Anti-HCV (positive/total, n)	2/15	2/8	
Best objective response (CR/PR/SD/PD, n)	0/5/10/0	0/0/0/8	
Median PFS (range, in month)	4.0 (1.5 – 36)	1.4 (1.0 – 4.0)	0.0313

The comparison between disease control and disease progression groups were performed by Mann-Whitney U test. AFP, α-fetoprotein; anti-HCV, anti-hepatitis C virus antibody; BCLC, Barcelona Clinic Liver Cancer; CR, complete response; HBsAg, hepatitis B viral antigen; PD, progression disease; PFS, progression free survival; PR, partial response; SD, stable disease.

### The axis of increased PD-1^+^ cells and decreased PD-L1^+^ cells were observed in advanced hepatocellular carcinoma patients carrying disease progression responses

**FIGURE 1:**
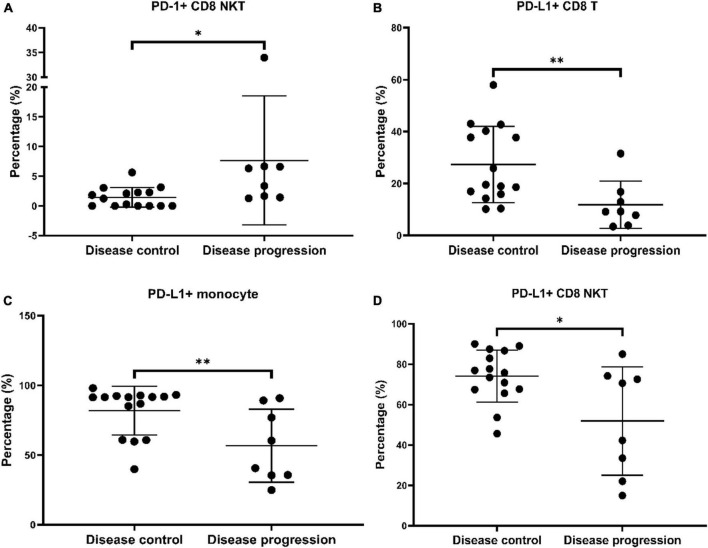
Axis of increased PD-1**^+^** cells and decreased PD-L1**^+^** cells was observed in advanced hepatocellular carcinoma (aHCC) patients carrying disease progression after nivolumab treatment. Peripheral blood mononuclear cells (PBMCs) from twenty-three aHCC patients were stained with targeted antibodies followed by analyzing the fluorescent pattern and used such pattern in gating immune cell subsets by flow cytometer, Kaluza analysis software, and definitive markers enlisted in Supplementary Table 1. The abundance of immune subsets in the subjects was measured, compared between the disease control and disease progression groups using Mann-Whitney U test, and shown in scatter plots with mean ± standard deviation (SD) by Prism. Four immune cell subsets, including PD-1 *^+^* CD8 NKT cells **(A)**, PD-L1***^+^*** CD8 T cells **(B)**, PD-L1***^+^*** monocytes **(C)**, and PD-L1***^+^*** CD8 NKT cells **(D)**, which abundances were significantly different between the disease control and disease progression groups. Immune subsets with statistical significance were labeled with * or ^**^ as *p* < 0.05 or *p* < 0.01.

We compared the percentages of fifty immune cell subsets between the disease control and disease progression groups to identify immune cell subsets with significantly different abundances. Higher percentages of PD-1^+^ CD8 NKTs were observed in the disease progression group than in the disease control group (Figure 1A). Additionally, lower percentages of PD-L1^+^ CD8 T cells, monocytes, and CD8 NKTs were found in the disease progression group than in the disease control group (Figures 1B–D). Altogether, the increased percentages of PD-1^+^ cells and decreased percentages of PD-L1^+^ cells were observed in aHCC patients with disease progression response after nivolumab treatment.

### Machine learning algorithm identified five immune subsets for discriminating advanced hepatocellular carcinoma patients with disease control disease progression responses

**FIGURE 2:**
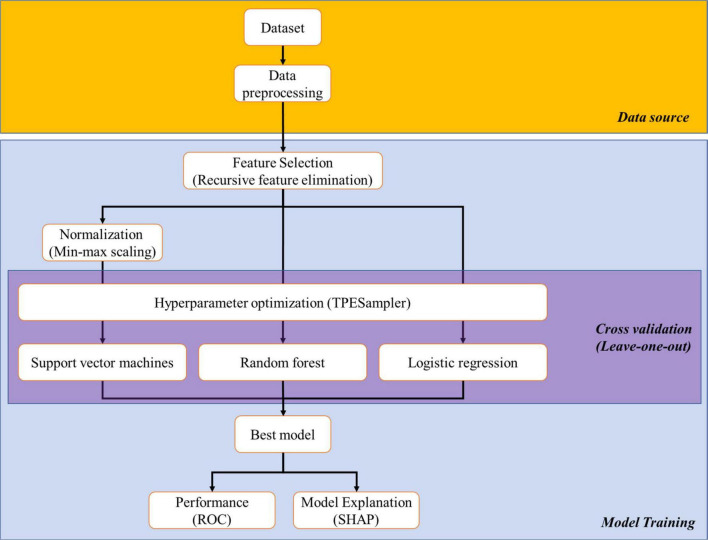
Schematic illustration of the process of machine learning and artificial intelligence. We collected data from all subjects (dataset) and removed the string variables inside (cleaned data). Featured subsets which were critical for distinguishing “disease control group” and “disease progression group” were selected from cleaned data by recursive feature elimination with cross validation (RFECV). Then, featured subsets proceeded with normalization (for support vector machines), hyperparameter optimization to determine the optimal parameters among three machine learning (ML) algorithms, and model training. Finally, the performance of the trained ML models and the ranking of the featured subsets among the models were carried out by receiver operation characteristic (ROC) and SHapley Additive exPlanations (SHAP), respectively.

To integrally analyze immunoprofiling, we constructed an ML platform to discriminate the disease control and the disease progression groups. As shown in Figure 2, the ML platform comprised one feature selection method (RFECV), one hyperparameter optimization method (Optuna), three ML algorithms (random forest classifier, logistic regression, support vector machines), and one model explanation method (SHAP).

**FIGURE 3:**
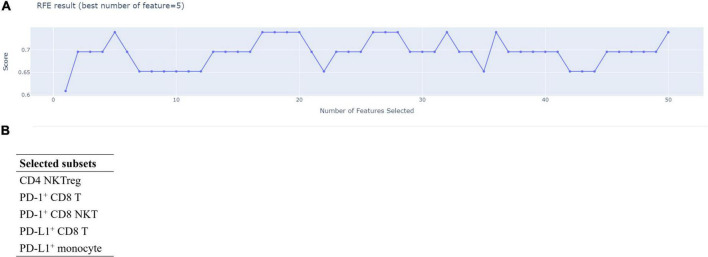
Five featured immune cell subsets had good performance in discriminating disease control and disease progression groups. A total of fifty immune cell subsets were screened by Recursive Feature Elimination with Cross-Validation (RFECV) to determine the number and the members of featured immune cell subsets in discriminating the disease control and disease progression groups. The screening result of RFECV were represented in the weighted average F1-score versus number of cell population subsets **(A)**. The highest score (0.7931) of average F1-score appeared while numbers of selected immune cell subsets was five. Therefore, the number featured immune cell subsets was five, and the featured immune cell subsets were enlisted in **(B)**.

We used RFECV to determine the minimal number of the featured immune cell subsets for discriminating the disease control and the disease progression groups and the members of featured immune cell subsets among the fifty immune cell subsets. As shown in Figure 3A, RFECV analysis got the highest score at five selected features, indicating that five immune cell subsets were enough to distinguish the disease control group from the disease progression group. The five featured immune cell subsets selected by RFECV were regulatory CD4 NKTs (CD4 NKTreg), PD-1^+^ CD8 T cells, PD-1^+^ CD8 NKTs, PD-L1^+^ CD8 T cells, and PD-L1^+^ monocytes (Figure 3B). Via the Mann-Whitney U test, we observed that aHCC patients carrying disease progression response to nivolumab treatment exhibited an immunoprofiling of lower percentages of PD-L1^+^ CD8 T cells, PD-L1^+^ monocytes, and higher percentages of PD-1^+^ CD8 NKTs (Figures 1A–C). These two results indicated that increased PD-L1 positivity on CD8 T cells and monocytes and decreased PD-1 positivity on CD8 NKTs might render aHCC patients more sensitive to nivolumab treatment.

### Five selected features efficiently distinguished advanced hepatocellular carcinoma patients with disease control response from those with disease progression response

**FIGURE 4:**
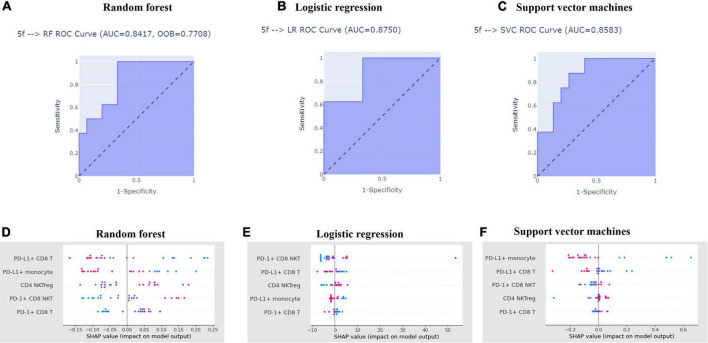
Machine learning algorithms efficiently distinguished aHCC patients with disease control responses from those with disease progression responses. Featured subsets for distinguishing disease control and disease progression groups were selected by recursive feature elimination and used to train the three machine learning models, which detailed procedure was described in Materials and Methods. After model training, the performance of random forest **(A)**, logistic regression **(B)**, and support vector machines **(C)** in distinguishing the disease control and disease progression groups were tested receiver operating characteristics. Also, the contribution of each featured subset in distinguishing disease control and disease progression groups model training among random forest **(D)**, logistic regression **(E)**, and support vector machines **(F)** was ranked by SHapley Additive exPlanations (SHAP), respectively.

We applied five selected features for training three ML algorithms (random forest classifier, logistic regression, support vector machines) and determined their performance of discriminating aHCC patients with disease control and disease progression responses via ROC analysis. aCCAAfter The AUC of ROC analysis among the three ML algorithms was 0.8417 (random forest classifier; Figure 4A), 0.8583 (logistic regression; Figure 4B), and 0.8750 (support vector machines; Figure 4C) respectively. This result indicated that the performance of the three ML models in discriminating disease control and disease progression groups was excellent (19). To evaluate the contribution of the five featured immune cell subsets within each ML algorithm, SHAP was used to rank the importance of the five featured immune cell subsets among the three ML algorithms. As shown in Figures 4D–F, SHAP values of the disease control and disease progression groups were distributed on the different sides of the Y-axis without overlapping, respectively. PD-L1^+^ CD8 T cells and PD-L1^+^ monocytes got the highest rank from the random forest and the logistic regression (Figures 4D,E). PD-1^+^ CD8 NKTs and PD-L1^+^ CD8 T cells got the highest rank in support vector machines (Figure 4F). Collectively, PD-L1^+^ CD8 T cells were highly ranked in the three ML algorithms, which pointed out that PD-L1^+^ CD8 T cells were highly critical in distinguishing the disease control group from the disease progression group. In summary, the ML platform efficiently discriminated the disease control group from the disease progression group, and the five featured immune cell subsets applied in the ML algorithms highly overlapped with the results of the Mann-Whitney U test.

## Discussion

In this study, significantly lower percentages of PD-L1^+^ CD8 T cells, monocytes, CD8 NKTs, and higher amounts of PD-1^+^ CD8 NKT were observed in aHCC patients with disease progression response than those with disease control response after nivolumab treatment via Mann-Whitney U test. Through RFECV method, five featured immune cell subsets, including CD4 NKTreg, PD-1^+^ CD8 NKTs, PD-1^+^ CD8 T cells, PD-L1^+^ CD8 T cells, and CD-L1^+^ monocytes, were selected from analyzed fifty immune cell subsets for ML training and been highly overlapping with ones identified by Mann-Whitney U test. This result implied that altering amounts of PD-1^+^ CD8 NKTs, PD-L1^+^ CD8 T cells, and CD-L1^+^ monocytes in peripheral blood might change the susceptibility of aHCC patients to nivolumab treatment. Trained ML algorithms committed valuable AUC from 0.8417 to 0.875 to significantly separate the disease control group from the disease progression group, and SHAP value ranking also revealed that PD-L1^+^ monocytes and PD-L1^+^ CD8 T cells exclusively contributed to this discrimination. Altogether, dissecting immunoprofiling with ML algorithms is promising for accurately predicting clinical responses of aHCC patients before receiving nivolumab treatment.

Several biomarkers, such as intratumoral expression of tumor neoantigens and PD-L1, tumor mutation burden, and DNA damage response pathway, are proposed to predict clinical responses to ICI treatments (20). However, the correlation between these predictive biomarkers and clinical outcomes of aHCC patients with ICI treatment is not strong (21), which indicates that selecting aHCC patients suitable for ICI treatment is still an unmet medical need. Several studies have revealed that altered amounts of specific immune cell subsets are associated with the drug responses of ICI treatment (22). Therefore, predictive biomarkers in the immune-cell profiling used to predict clinical responses of ICI treatment for aHCC patients are promising. HCC patients who got clinical benefits from ICI treatment exhibited higher percentages of CD4 effector memory T cells, CD8 effector memory T cells, PD-1^+^ CD8 T cells, HLA-DR+ dendritic cells (DCs), lower amounts of CD14^+^ MDSCs, CD14^+^CD16^–^ and CD14^+^CD16^+^ monocytes than whoever did not get clinical benefit from ICI treatment (23, 24). Our previous study indicated that aHCC patients with disease progression response carried an immunoprofiling of higher percentages of PD-1^+^ B cells (8). After enrolling additional aHCC patients and modifying the repertoire of analyzed immune cell subsets, we further observed that aHCC patients carrying disease progression response exhibited decreased positivity of PD-L1 on CD8 T cells, monocytes, CD8 NKTs, and increased positivity of PD-1 on CD8 NKTs (Figure 1). The five immune cell subsets we identified could be applied in selecting aHCC patients suitable for nivolumab treatment.

In the current study, we identified augmented percentages of PD-1^+^ NKTs in the aHCC patients with disease progression response than those in the disease control response (Figure 1A). Non-small-cell lung cancer and advanced melanoma patients carrying increased PD-1^+^ NKTs exhibited shorter OS after nivolumab treatment (25, 26). The increased percentages of PD-1^+^ NKTs were under exhausting conditions and could be invigorated by anti-PD-L1 antibodies (27, 28). These results implied that anti-PD-L1 antibodies such as atezolizumab might benefit aHCC patients with disease progression response to anti-PD-1 treatment in the clinics.

Additionally, lower percentages of PD-L1^+^ CD8 T cells were identified in the aHCC patients with disease progression response than those with the disease control response (Figure 1B). Lower positivity of PD-L1 on T cells (including CD4 T cells and CD8 T cells) and monocytes were linked to shorter OS in patients with various cancers after ICI treatments (mainly PD-1 inhibitors) (29, 30). Of note, Jacquelot et al. reported that melanoma patients who got clinical benefits from ipilimumab (CTLA-4 inhibitor) had lower positivity of PD-L1 on CD8 T cells than whoever did not get clinical benefits from ipilimumab (31). These two studies reveal that melanoma patients with PD-1 inhibitors treatment failure may get clinical benefits from ipilimumab treatment, and the case report published by Sakai et al. confirmed this concept (32). The current study was the first report addressing the correlation between disease progression response after nivolumab treatment in aHCC patients and the lower positivity of PD-L1 on CD8 T cells, and those aHCC patients with disease progression response may get clinical benefits from ipilimumab treatment.

We reported the first study attempting to integrally parse the immunoprofiling of aHCC using ML algorithms. Zhou et al. use a random forest classifier and Least absolute shrinkage and selection operator (LASSO)-Cox regression and determine that increased neutrophils, CD8 T cells, but decreased plasmacytoid DCs, monocytes, and NKT are linked to shorter OS in cancer patients after ICI treatment (33). Additionally, Peng et al. use RFE and LASSO to find immune subsets correlating with the major-pathological responses (MPR) of ICI treatment for NSCLC patients. In their study, increases in NKTs, CD4^+^CD45RA^–^ T cells, but decreases in B cells and CD4^+^CD45RA^+^ T cells are associated with MPR after ICI treatment (34). These studies indicate that the ML algorithms can help us identify cancer patients suitable for ICI treatment through the hidden patterns parsing from immunoprofiling. We identified five featured immune cell subsets (CD4 NKTreg, PD-1^+^ CD8 T cells, PD-1^+^ CD8 NKTs, PD-L1^+^ CD8 T cells, and PD-L1^+^ monocytes) that applied in ML model training via RFECV (Figure 3B). ML algorithms significantly discriminated the disease-control and the disease-progression groups after training with the five featured immune cell subsets (Figure 4). These results reinforce the feasibility of applying the ML algorithm in parsing immunoprofiling from aHCC. Furthermore, significant decreases in PD-L1^+^ CD8 T cells and monocytes but increases in PD-1^+^ CD8 NKTs were observed in aHCC patients carrying disease progression response (Figures 1A–C). This result implied that changing the positivity of PD-1 and PD-L1 on definite immune cell subsets in peripheral blood might alter the susceptibility of aHCC patients to ICI treatment.

## Conclusion

Our preliminary investigation posed that applying computational analysis in dissecting sophisticated immunoprofiling and serving as predictive biomarkers of ICI treatment for aHCC patients is feasible. Furthermore, the changing of PD-1 and PD-L1 expression upon definite immune cell subsets may correlate to the susceptibility of aHCC to nivolumab treatment. Although the results of the current study were promising for predicting the treatment response of immune checkpoint inhibitors (ICIs), several studies reported some limitations in machine learning applications, such as overfitting (35). To attenuate the risk of overfitting and promote the performance, we applied the recursive feature elimination with cross-validation (RFECV) and leave-one-out methods in this study (36, 37). Nevertheless, the small sample size and lack of external validation still caused the inevitable risk of overfitting in the current study. We continuously collected new data for model refinement and external validation.

## Data availability statement

The original contributions presented in this study are included in the article/Supplementary material, further inquiries can be directed to the corresponding author.

## Ethics statement

The studies involving human participants were reviewed and approved by the Institution Review Board of Taipei Veterans General Hospital. The patients/participants provided their written informed consent to participate in this study.

## Author contributions

Y-PH and J-ML conceived the study and reviewed the draft. Y-PH supervised the enrollment of the subjects and reviewed the draft. K-YC performed the investigation of immunoprofiling and statistical analysis. C-YL constructed the machine learning platform and performed the investigation. S-RL wrote the original draft. Y-HT, W-YL, Y-YS, CH, C-HH, and YC managed the enrollment of the subjects. All authors had reviewed the manuscript and approved that publication of the manuscript.

## Footnotes

1
https://github.com/optuna/optuna

2
https://github.com/slundberg/shap
