# Supplementary material for: Artificial intelligence-based immunoprofiling serves as a potentially predictive biomarker of nivolumab treatment for advanced hepatocellular carcinoma
Source: Front Med (Lausanne). 2022 Nov 8;9:1008855. doi: 10.3389/fmed.2022.1008855 (PMC9679144; doi:10.3389/fmed.2022.1008855)
Supplement: Supplementary file 1 [file Data_Sheet_1.docx]

**Supplementary Table 1.** Phenotypes of immune cell subsets analyzed in this study

| Cell name | Markers |
| --- | --- |
| *NK cell* |  |
| NK | CD3^-^CD14^-^CD19^-^CD56^+^ |
| CD8 NK | CD3^-^CD14^-^CD19^-^CD56^+^CD4^-^CD8^+^ |
| DN NK | CD3^-^CD14^-^CD19^-^CD56^+^CD4^-^CD8^-^ |
| *NKT cell* |  |
| NKT | CD3^+^CD14^-^CD19^-^CD56^+^ |
| CD4 NKT | CD3^+^CD14^-^CD19^-^CD56^+^CD4^+^CD8^-^ |
| CD8 NKT | CD3^+^CD14^-^CD19^-^CD56^+^CD4^-^CD8^+^ |
| *Dendritic cell* |  |
| DC | CD3^-^CD14^-^CD19^-^CD56^-^CD11c^+^ |
| *B cell* |  |
| B | CD3^-^CD14^-^CD19^+^CD56^-^ |
| *Monocyte* |  |
| Monocyte | CD3^-^CD14^+^CD19^-^CD56^dim^ |
| *T lymphocyte* |  |
| Total T | CD3^+^CD14^-^CD19^-^CD56^-^ |
| CD4 αβ T | CD3^+^CD14^-^CD19^-^CD56^-^TCR_αβ_^+^TCR_γδ_^-^CD4^+^CD8^-^ |
| CD25^-^CD69^-^CD4 αβ T | CD3^+^CD14^-^CD19^-^CD56^-^TCR_αβ_^+^TCR_γδ_^-^CD4^+^CD8^-^CD25^-^CD69^-^ |
| CD25^+^CD69^-^CD4 αβ T | CD3^+^CD14^-^CD19^-^CD56^-^TCR_αβ_^+^TCR_γδ_^-^CD4^+^CD8^-^CD25^+^CD69^-^ |
| CD25^-^CD69^+^CD4 αβ T | CD3^+^CD14^-^CD19^-^CD56^-^TCR_αβ_^+^TCR_γδ_^-^CD4^+^CD8^-^CD25^-^CD69^+^ |
| CD8 αβ T | CD3^+^CD14^-^CD19^-^CD56^-^TCR_αβ_^+^TCR_γδ_^-^CD4^-^CD8^+^ |
| CD25^-^CD69^-^CD8 αβ T | CD3^+^CD14^-^CD19^-^CD56^-^TCR_αβ_^+^TCR_γδ_^-^CD4^-^CD8^+^CD25^-^CD69^-^ |
| CD25^+^CD69^-^CD8 αβ T | CD3^+^CD14^-^CD19^-^CD56^-^TCR_αβ_^+^TCR_γδ_^-^CD4^-^CD8^+^CD25^+^CD69^-^ |
| CD25^-^CD69^+^CD8 αβ T | CD3^+^CD14^-^CD19^-^CD56^-^TCR_αβ_^+^TCR_γδ_^-^CD4^-^CD8^+^CD25^-^CD69^+^ |
| CD8 γδ T | CD3^+^CD14^-^CD19^-^CD56^-^TCR_αβ_^-^TCR_γδ_^+^CD4^-^CD8^+^ |
| DN γδ T | CD3^+^CD14^-^CD19^-^CD56^-^TCR_αβ_^-^TCR_γδ_^+^CD4^-^CD8^-^ |
| *PD-1^+^ cells* |  |
| PD-1^+^ NK | CD3^-^CD14^-^CD19^-^CD56^+^PD-1^+^PD-L1^-^ |
| PD-1^+^ CD4 NKT | CD3^+^CD14^-^CD19^-^CD56^+^CD4^+^CD8^-^PD-1^+^PD-L1^-^ |
| PD-1^+^ CD8 NKT | CD3^+^CD14^-^CD19^-^CD56^+^CD4^-^CD8^+^PD-1^+^PD-L1^-^ |
| PD-1^+^ DC | CD3^-^CD14^-^CD19^-^CD56^-^CD11c^+^PD-1^+^PD-L1^-^ |
| PD-1^+^ monocyte | CD3^-^CD14^+^CD19^-^CD56^dim^PD-1^+^PD-L1^-^ |
| PD-1^+^ B | CD3^-^CD14^-^CD19^+^CD56^-^PD-1^+^PD-L1^-^ |
| PD-1^+^ CD4 T | CD3^+^CD14^-^CD19^-^CD56^-^CD4^+^CD8^-^PD-1^+^PD-L1^-^ |
| PD-1^+^ CD8 T | CD3^+^CD14^-^CD19^-^CD56^-^CD4^-^CD8^+^PD-1^+^PD-L1^-^ |
| *PD-L1^+^ cells* |  |
| PD-L1^+^ NK | CD3^-^CD14^-^CD19^-^CD56^+^PD-1^-^PD-L1^+^ |
| PD-L1^+^ CD4 NKT | CD3^+^CD14^-^CD19^-^CD56^+^CD4^+^CD8^-^PD-1^-^PD-L1^+^ |
| PD-L1^+^ CD8 NKT | CD3^+^CD14^-^CD19^-^CD56^+^CD4^-^CD8^+^PD-1^-^PD-L1^+^ |
| PD-L1^+^ DC | CD3^-^CD14^-^CD19^-^CD56^-^CD11c^+^PD-1^-^PD-L1^+^ |
| PD-L1^+^ monocyte | CD3^-^CD14^+^CD19^-^CD56^dim^PD-1^-^PD-L1^+^ |
| PD-L1^+^ B | CD3^-^CD14^-^CD19^+^CD56^-^PD-1^-^PD-L1^+^ |
| PD-L1^+^ CD4 T | CD3^+^CD14^-^CD19^-^CD56^-^CD4^+^CD8^-^PD-1^-^PD-L1^+^ |
| PD-L1^+^ CD8 T | CD3^+^CD14^-^CD19^-^CD56^-^CD4^-^CD8^+^PD-1^-^PD-L1^+^ |
| *PD1^+^PD-L1^+^ cells* |  |
| PD-1^+^PD-L1^+^ NK | CD3^-^CD14^-^CD19^-^CD56^+^PD-1^+^PD-L1^+^ |
| PD-1^+^PD-L1^+^ CD4 NKT | CD3^+^CD14^-^CD19^-^CD56^+^CD4^+^CD8^-^PD-1^+^PD-L1^+^ |
| PD-1^+^PD-L1^+^ CD8 NKT | CD3^+^CD14^-^CD19^-^CD56^+^CD4^-^CD8^+^PD-1^+^PD-L1^+^ |
| PD-1^+^PD-L1^+^ DC | CD3^-^CD14^-^CD19^-^CD56^-^CD11c^+^PD-1^+^PD-L1^+^ |
| PD-1^+^PD-L1^+^ monocyte | CD3^-^CD14^+^CD19^-^CD56^dim^PD-1^+^PD-L1^+^ |
| PD-1^+^PD-L1^+^ B | CD3^-^CD14^-^CD19^+^CD56^-^PD-1^+^PD-L1^+^ |
| PD-1^+^PD-L1^+^ CD4 T | CD3^+^CD14^-^CD19^-^CD56^-^CD4^+^CD8^-^PD-1^+^PD-L1^+^ |
| PD-1^+^PD-L1^+^ CD8 T | CD3^+^CD14^-^CD19^-^CD56^-^CD4^-^CD8^+^PD-1^+^PD-L1^+^ |
| *Regulatory cells* |  |
| CD4 Treg | CD3^+^CD14^-^CD19^-^CD56^-^CD4^+^CD8^-^FoxP3^+^CD25^+^ |
| CD8 Treg | CD3^+^CD14^-^CD19^-^CD56^-^CD4^-^CD8^+^FoxP3^+^CD25^+^ |
| CD8 NKreg | CD3^-^CD14^-^CD19^-^CD56^+^CD4^-^CD8^+^FoxP3^+^CD25^+^ |
| DN NKreg | CD3^-^CD14^-^CD19^-^CD56^+^CD4^-^CD8^-^FoxP3^+^CD25^+^ |
| CD4 NKTreg | CD3^+^CD14^-^CD19^-^CD56^+^CD4^+^CD8^-^FoxP3^+^CD25^+^ |
| CD8 NKTreg | CD3^+^CD14^-^CD19^-^CD56^+^CD4^-^CD8^+^FoxP3^+^CD25^+^ |

**Abbreviation:** DC, dendritic cell; DN, double negative; MHC II, major histocompatibility complex class II; NK, natural killer cell; NKT, natural killer T cell; PD-1, programmed cell death 1; PD-L1, programmed cell death ligand 1; TCR, T cell receptor.

**Supplementary Table 2**. Hyperparameters applied in the machine learning algorithms

| **Hyperparameter** | **Value** | **Discription** |
| --- | --- | --- |
| *Random_Forest* | | |
| n_estimators | 500 | The number of decision tree to be created in model. |
| max_depth | 4 | The maximum depth of the tree. |
| min_samples_split | 3 | The minimum numbers of samples required to split an internal node. |
| random_state | 15 | Controls both the randomness of the bootstrapping of the samples used when building trees  and the sampling of the features to consider when looking for the best split at each node. |
| criterion | entropy | The function to measure the quality of a split. |
| oob_score | True | Whether to use out-of-bag samples to estimate the generalization score. |
|  |  |  |
| *Logistic Regression* | | |
| solver | liblinear | Algorithm to use in the optimization problem. |
| C | 5.4 | Inverse of regularization strength. |
|  |  |  |
| *Support Vector Machines* | | |
| C | 2.1 | The strength of the regularization is inversely proportional to C. |
| gamma | 0.21 | Kernel coefficient |


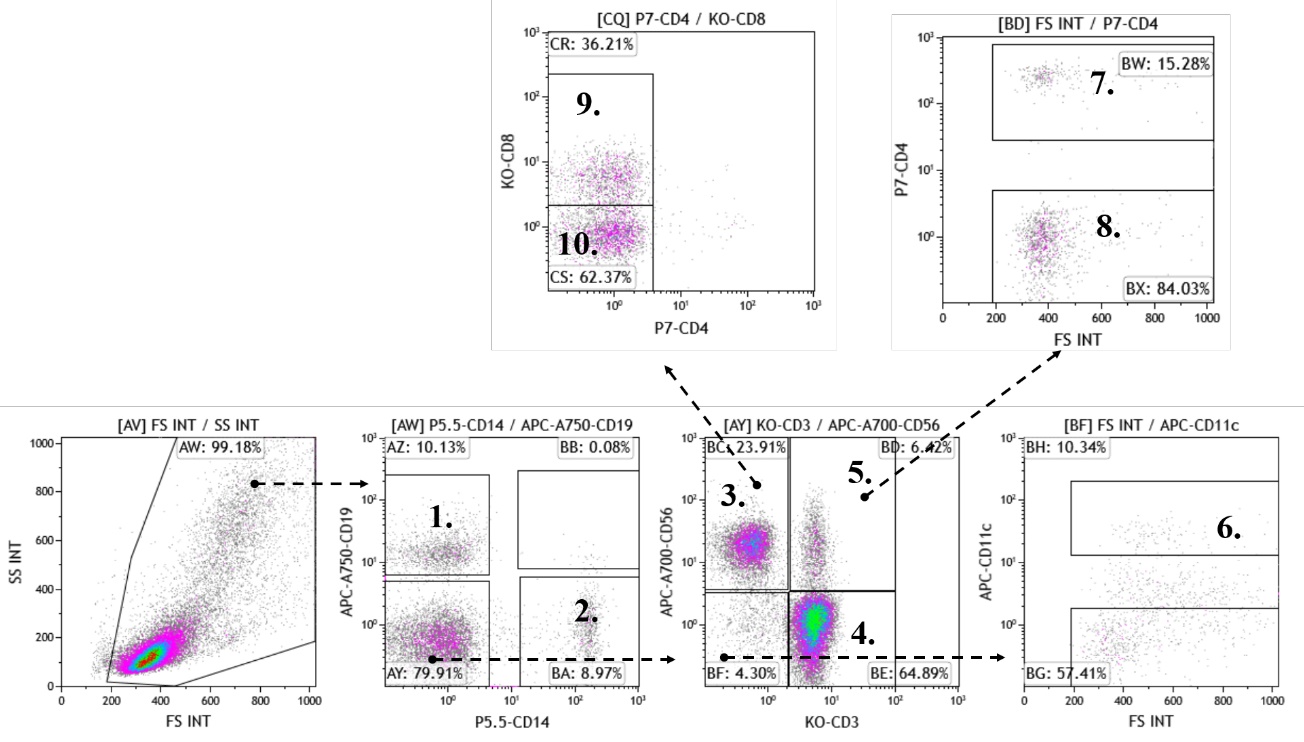


**Figure S1. Representative illustration of gating process of lineage cells.**

PBMCs were firstly gated with CD14/CD19 to identify B cells (**1**) and monocytes (**2**). Then, the CD14^-^CD19^-^ subpopulation was gated with CD3 and CD56 for NK cells (**3**), T cells (**4**), and NKT cells (**5**). Dendritic cells (DCs; **6**) were gated from CD3^-^CD56^-^ subpopulation by CD11c. Meanwhile, NK and NKT cells were further divided into CD4 NKT (**7**), CD8 NKT (**8**), CD8 NK (**9**), and DN NK cells (**10**) using CD4 and CD8, respectively.


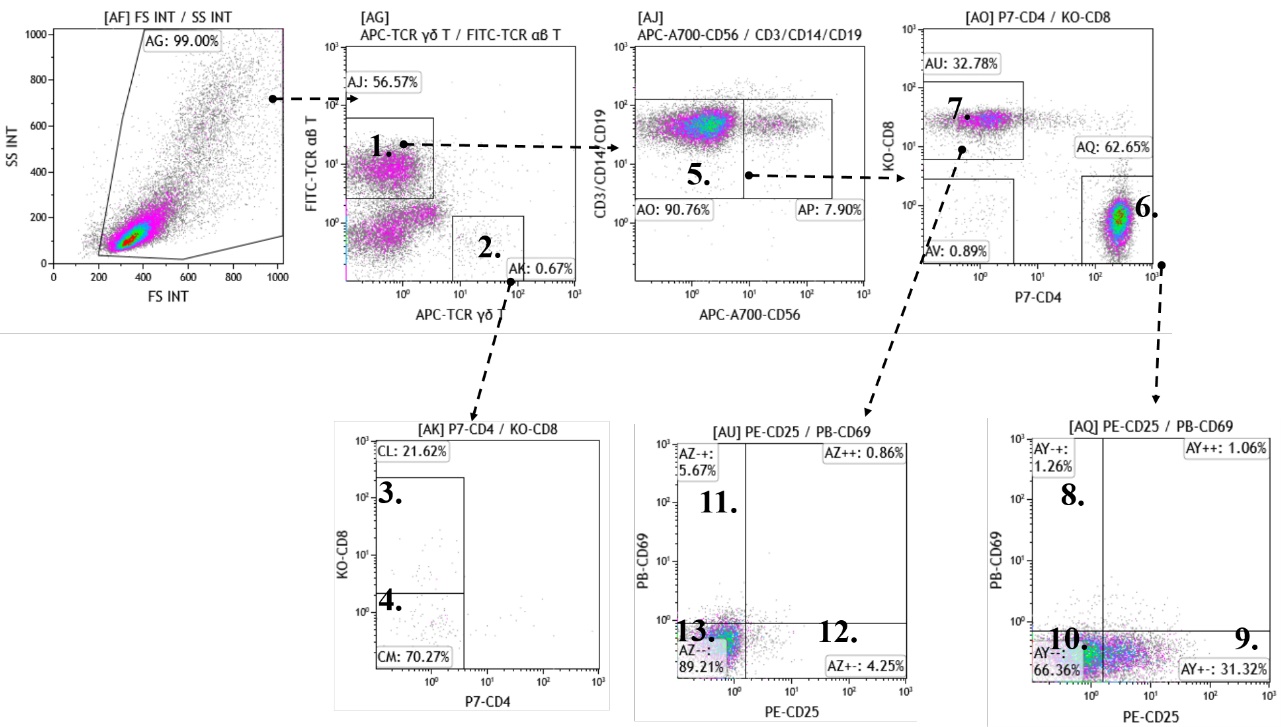


**Figure S2. Representative illustration of gating procedure of T cells**

PBMCs were gated with TCRαβ TCRγδ to identify TCRαβ^+^ cells (**1**) and TCRγδ^+^ cells (**2**). TCRγδ^+^ cells were gated by CD4 and CD8 to identify CD8 γδT cells (**3**) and DN γδT cells (**4**). Meanwhile, the CD3^+^CD56^-^ subpopulation within TCRαβ^+^ cells was defined αβ T cells (**5**) and sequentially gated with CD4/CD8 to identify CD4 αβT cells (**6**) and CD8 αβT cells (**7**); and CD25^-^CD69^+^CD4 αβT cells (**8**), CD25^-^CD69^+^CD8 αβT cells (**11**), CD25^+^CD69^-^CD4 αβT cells (**9**), CD25^+^CD69^-^CD8 αβT cells (**12**), CD25^-^CD69^-^CD4 αβT cells (**10**), and CD25^-^CD69^-^CD8 αβT cells (**13**) by CD25 and CD69, respectively.


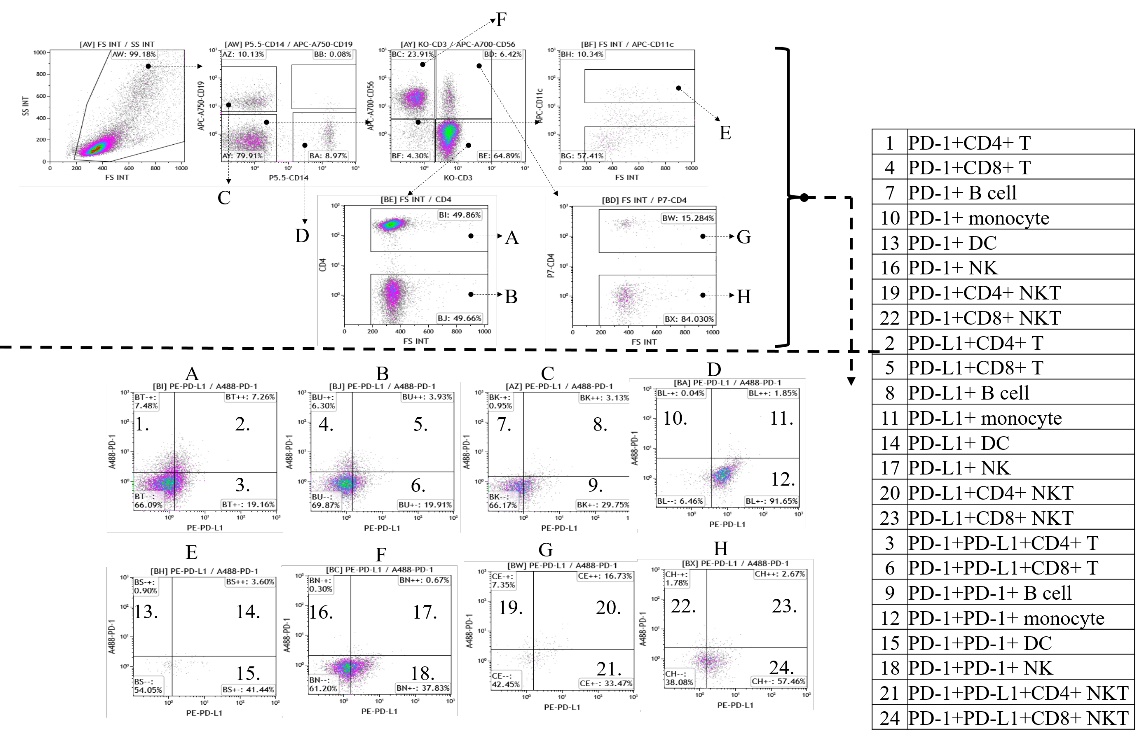


**Figure S3. Representative illustration of the gating procedure of PD-1^+^ and PD-L1^+^ cells.**

Lineage cells including CD4 T cells (**A**), CD8 T cells (**B**), B cells (**C**) monocytes, (**D**) dendritic cells (DCs), (**E**) natural killer cells (NKs) (**F**), CD4 NKT cells (**G**), and CD8 NKT cells (**H**) were gated based on the description in Materials and Methods. Then, lineage cells were grouped into PD-1^+^ cells, PD-L1^+^ cells, and PD-1^+^PD-L1^+^ cells by gating with PD-1 and PD-L1.


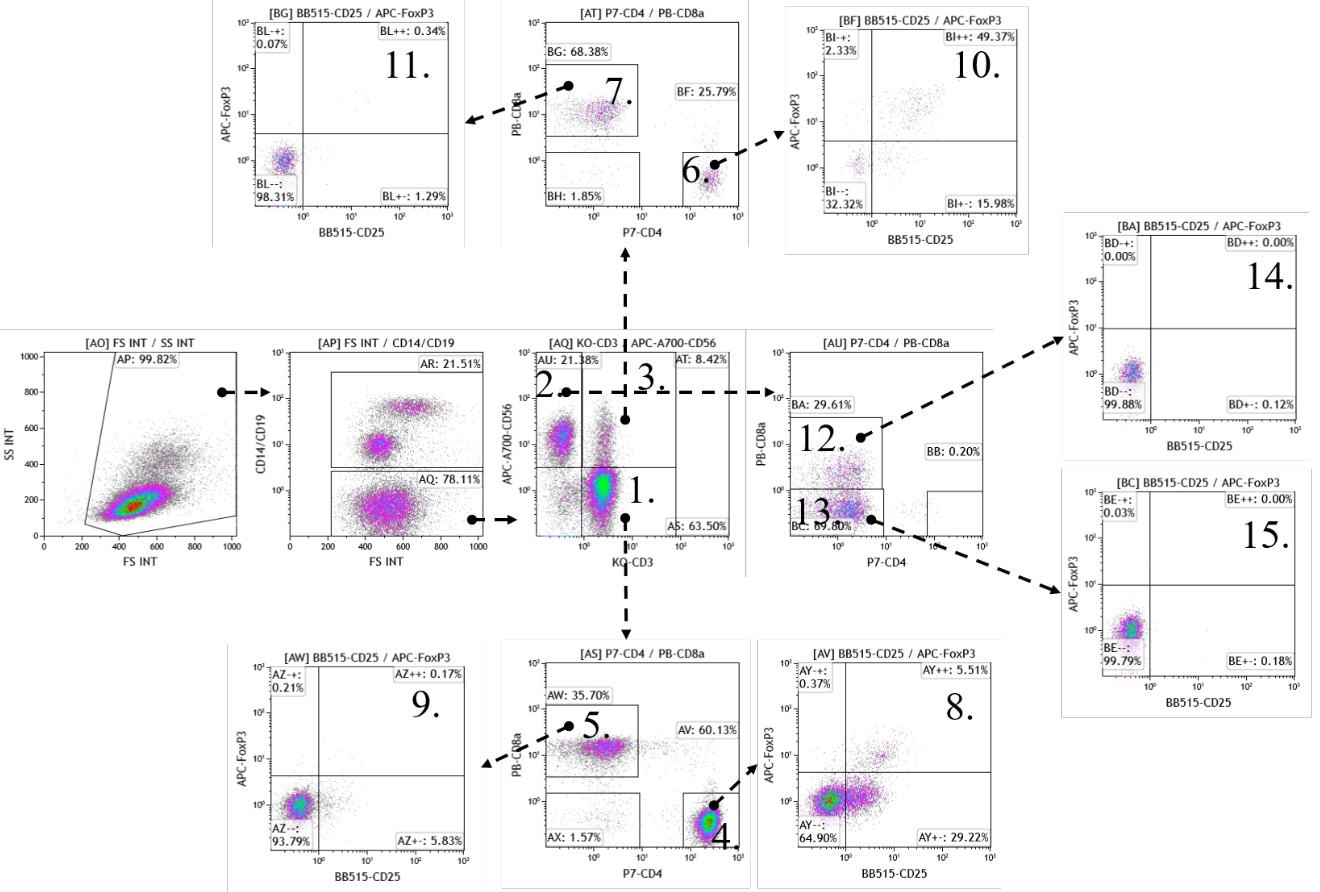


**Figure S4. Representative illustration of gating procedure of regulatory cells.**

PBMCs were grouped into T cells (1), NK cells (2), and NKT cells (3). T cells and NKT cells were firstly divided into CD4 T (**4**), CD4 NKT (6), CD8 T (5), and CD8 NKT cells (7), followed by defining FoxP3^+^CD25^+^ subpopulations as CD4 Treg (**8**), CD4 NKTreg (**10**), CD8 Treg (**9**), and CD8 NKTreg (**11**), respectively. NK cells were separated into CD8 NK (**12**) and DN NK (**13**), and the corresponding subpopulations with FoxP3^+^CD25^+^ phenotypes were defined as CD8 NKreg (**14**) and DN NKreg (**15**).
